# Development of a quantitative pachytene chromosome map and its unification with somatic chromosome and linkage maps of rice (*Oryza sativa* L.)

**DOI:** 10.1371/journal.pone.0195710

**Published:** 2018-04-19

**Authors:** Nobuko Ohmido, Aiko Iwata, Seiji Kato, Toshiyuki Wako, Kiichi Fukui

**Affiliations:** 1 Graduate School of Human Development and Environment, Kobe University, Kobe, Hyogo, Japan; 2 Center for Applied Genetic Technologies, University of Georgia, Athens, Georgia, United States of America; 3 Yamanashi Prefectural Agritechnology Center, 1100, Shimoimai, Kai, Yamanashi, Japan; 4 Advanced Analysis Center, National Agriculture and Food Research Organization, Tsukuba, Ibaraki, Japan; 5 Graduate School of Pharmaceutical Sciences, Osaka University, Suita, Osaka, Japan; The Institute of Genetics and Developmental Biology (IGDB) of the Chinese Academy of Sciences (CAS), China, CHINA

## Abstract

A quantitative pachytene chromosome map of rice (*Oryza sativa* L.) was developed using imaging methods. The map depicts not only distribution patterns of chromomeres specific to pachytene chromosomes, but also the higher order information of chromosomal structures, such as heterochromatin (condensed regions), euchromatin (decondensed regions), the primary constrictions (centromeres), and the secondary constriction (nucleolar organizing regions, NOR). These features were image analyzed and quantitatively mapped onto the map by Chromosome Image Analyzing System ver. 4.0 (CHIAS IV). Correlation between H3K9me2, an epigenetic marker and formation and/or maintenance of heterochromatin, thus was, clearly visualized. Then the pachytene chromosome map was unified with the existing somatic chromosome and linkage maps by physically mapping common DNA markers among them, such as a rice A genome specific tandem repeat sequence (TrsA), 5S and 45S ribosomal RNA genes, five bacterial artificial chromosome (BAC) clones, four P1 bacteriophage artificial chromosome (PAC) clones using multicolor fluorescence *in situ* hybridization (FISH). Detailed comparison between the locations of the DNA probes on the pachytene chromosomes using multicolor FISH, and the linkage map enabled determination of the chromosome number and short/long arms of individual pachytene chromosomes using the chromosome number and arm assignment designated for the linkage map. As a result, the quantitative pachytene chromosome map was unified with two other major rice chromosome maps representing somatic prometaphase chromosomes and genetic linkages. In conclusion, the unification of the three rice maps serves as an indispensable basic information, not only for an in-depth comparison between genetic and chromosomal data, but also for practical breeding programs.

## Introduction

Chromosome map is a geographic representation of chromosomes within a genome in which their relative sizes are drawn and genes are plotted according to, either their relative or absolute distance. There are two kinds of chromosome maps—genetic and cytological maps, whose basis are the recombination values of the genes and physical length of the mitotic or meiotic chromosomes, respectively. Chromosome maps have been constructed throughout the 20^th^ century, based on both the facts that chromosomes could be observed as lod/dot like objects under a microscope, and that the genes are linearly arranged on the chromosome. Given that the chromosome maps present the geometry of genetic information on an individual chromosome, both maps are useful for genetic research and breeding programs, like to a road map of the navigator system used in automobiles. Analogous to the travel situation, different road maps represent varying distances to the destination, duration, and travel costs, etc., each chromosome map has its own geometric representation of biological information. Therefore, it would be ideal if the different chromosome maps are unified, and present diverse information in an easy and comparable form. However, there has been no quantitative chromosome map that represents meiotic pachytene chromosomes in rice (*Oryza sativa* L.), even though a quantitative mitotic chromosome map and detailed linkage map have already been developed by imaging of mitotic prometaphase chromosomes in 1991 [1–8].

As pachytene and somatic chromosome maps provide basic and useful information for genetic research, and designing breeding strategy, the former has been developed in many crops especially with small chromosomes, such as tomato [9], maize [10], *Medicago truncatula* [11]., and the latter in the crops with large chromosomes, such as barely [12–15], wheat [16, 17], rye [18, 19] etc. In rice, containing small chromosomes, pachytene chromosome maps have also been handwritten by experienced and skillful rice cytogeneticists and reported throughout the 20^th^ century [20–23]. Khush et al. [21] and Kurata et al. [23] depicted 300, 340 chromomeres in a total of 12 rice chromosomes, respectively, indicating the use of longer pachytene chromosomes than what we used in this study. Although, these maps showed the similar characteristics among them, they have little compatibility with the recent quantitative genetic and chromosome maps. In the absence of quantitative data in the scale of figures, or number of the chromosomes used to develop the individual chromosome maps, compatibility can be challenging. Some handwritten somatic chromosome maps are also available to date [23], and the situation with these maps are similar to the pachytene chromosome maps, i.e., they are similar to each other, but difficult to be used in a comparable way. Linking the rice linkage map and the mitotic chromosome map was explored by using trisomic series [21, 24].

Chromosome dynamics in their structures through M phase have been thoroughly investigated, mainly because they are visible as biological objects by optical microscopy. Morphological features, such as banding patterns appearing on the chromosomes are the important markers for chromosome identification and mapping genes [12, 13, 25]. Imaging is an effective method to analyze chromosome images quantitatively and dynamically. Chromosome imaging has a historical perspective over quarter of a century after the first versatile chromosome image analyzing system was developed [26, 27]. The number of rice chromosomes was determined as 2*n* = 24 in 1910 [28], and objective identification and quantitative characterization of the individual somatic rice chromosomes by using imaging methods followed in 1991, based on the accumulation of years of research work on rice chromosomes [1–3, 6, 8, 21, 22]. As it is entirely impossible to identify or characterize the individual rice chromosomes at the mitotic metaphase given their small dimension, a quantitative chromosome map was developed using mitotic chromosomes at the somatic prometaphase [1, 2]. Subsequently, an imaging method that enabled analysis of a rice pachytene chromosome, Chromosome 9, was developed [29]. Now a quantitative pachytene chromosome map covering all the twelve chromosomes has been developed using the same method. Furthermore, the pachytene chromosome map is unified with mitotic chromosome and linkage maps referencing the physical mapping data of the specific nucleotide sequences by multicolor fluorescence *in situ* hybridization (FISH). Unification of the different numbering systems independently developed among the mitotic chromosomes, pachytene chromosomes, and linkage groups has already been proposed [7]. Further annotation of short and long arms was demonstrated in 2005 by the Rice Genome Research Program (RGP) [30], based on the number of nucleotide sequences decoded for individual chromosomes, which covered 96.6–97.1% of the rice genome (384.2–386.5 Mb) in IRGSP 1.0, released at 2011 (http://rapdb.dna.affrc.go.jp/index.html) [31, 32]

Physical mapping of the genes/nucleotide sequences, and immunodetection of the proteins of interest on chromosomes have been performed by ISH/FISH and immunofluorescence staining, respectively [33–35]. They enable direct comparison between the locations of genes/marker sequences on pachytene/mitotic chromosomes, and their locations on the linkage map [36–42]. Distribution of the specific protein modification also indicates the status of chromatin, gene activity and the stage of cell cycle [43]. Identification of cytological features, for example, heterochromatin, euchromatin, and studying how these structures are formed during a cell cycle provides essential information on chromatin condensations, regulation of gene expression, and identification of the chromosomes. In case of small plant chromosomes such as rice, somatic prometaphase chromosomes show uneven condensation throughout the chromosome, which is reproducibly generated and visualized by Giemsa staining [1, 2]. In order to analyze cytological features of plant chromosomes in detail, such as rice, information with much higher resolution provided by a pachytene chromosome map also seems to be effective [42]. The heterochromatic regions in the pachytene chromosomes were determined based on the fluorescent intensity of 4’,6-diamidino-2-phenylindole (DAPI) and propidium iodide (PI) staining along the rice chromosome assuming that the regions with high fluorescent intensity correspond to the regions of high chromatin density.

To examine the possible mechanism of heterochromatin formation by histone modification, which is known to be involved in the epigenetic regulation of gene expression [44], and condensation of chromomeres as the distribution pattern of histone H3 di-methylation at lysine 9 (H3K9me2) were image-analyzed in detail. H3K9me2 is a well-known epigenetic modification for chromatin condensation, and thus the suppression of gene expression [43, 45]. Furthermore, to explore the possible causes of differences among existing rice pachytene chromosome maps, the condensation dynamics of pachytene chromosomes were also image-analyzed. As a result, each chromosomal region was revealed to have its own condensation dynamics, resulting its own stage specific lengths and variable chromomere distribution patterns even in the same chromosome when the sampling stages were different.

We report here, a standardized and complete rice pachytene chromosome map depicting cytological features with the positions of specific nucleotide sequences, validated by FISH and histone methylation by immunostaining. This map is anchored by centromere-specific and chromosomal arm-specific BAC/PAC clones, which were already mapped on the linkage map, and were used for integration between the maps [20]. Linkage maps based on recombination values do not directly reflect physical distances among genes. Therefore, unification of the four major maps representing nucleotide numbers, linkage groups, meiotic pachytene chromosomes and mitotic prometaphase chromosomes provides fundamental and basic information, both, for genetic studies and breeding practices.

## Materials and methods

### Plant materials and cytology

A rice cultivar, Nipponbare (*Oryza sativa* L. ssp. *japonica*, 2*n* = 2*x* = 24) was used throughout the study. Young panicles about 7–10 days before heading were harvested and fixed in a fixative solution (ethanol: acetic acid = 3:1). After rinsing in distilled water, anthers (0.66–0.76 mm) were excised, and squashed on a glass slide with 20% acetic acid. After freezing the slides on dry ice, the coverslips were removed. The slides were air-dried and stored at -20°C, until further use.

### Microscopy and image analyses

Chromosome images were directly captured through a cooled CCD camera (PXL1400, Photometrics, Tucson, AZ, USA) mounted on a fluorescence microscope (BX60, Olympus, Tokyo). The digitized images were analyzed using Chromosome image analyzing system ver. 4 (CHIAS IV) [46, 47], for which publicly available image processing software, ImageJ (http://rsb.info.nih.gov/ij/) was used. CHIAS IV is the latest version developed specifically for detailed analyses of pachytene chromosomes [26]. Detailed image analysis steps of pachytene chromosomes using CHIAS III were described previously [29, 48]. The CHIAS IV program and an instruction manual is available at: http://www2.kobe-u.ac.jp/~ohmido/index03.htm. We defined the parameters that represent chromatin compaction in pachytene chromosomes as the division of the estimated DNA content (Mb) of the region by the physical length in μm. Three fluorescence filters of U-MNIBA, U-MNG, and U-MNU (Olympus) were used for the detection of the individual fluorescence from fluorescein isothiocyanate (FITC), Cy3, and DAPI, respectively. Images were captured, merged and pseudocolored using IPLab Spectrum^™^ (Version 2.4).

### Fluorescence *in situ* hybridization (FISH)

The probes used for identification of individual chromosomes are listed in Table 1. Chromosome-specific BAC and PAC clones were selected from the map of RGP website (http://rapdb.dna.affrc.go.jp/) and the clones with ID No. starting with the letter P or B were obtained from the DNA bank of the National Institute of Agrobiological Science (NIAS), Japan. OSJNBa and OSJNBb clones were obtained from Clemson University Genomics Institute (CUGI), USA. Pericentromere-specific BAC clone, B1109A06, containing rice centromere-specific CentO sequence [49], chromosome-specific BAC/PAC, 45S ribosomal RNA (rDNA) gene [50, 51], 5S rDNA [14], and A-genome-specific tandem repeat sequence, TrsA [52, 53] were used as landmarks for the specific regions of the individual chromosomes. The 45S rDNA and BAC/PAC clones were labeled with biotin-16dUTP or digoxigenin-11dUTP (Roche, Branchburg, NJ, USA) using a Nick Translation Kit (Roche). 5S rDNA gene and TrsA were amplified and labeled with biotin-16dUTP or digoxigenin-11dUTP by PCR [54]. The FISH procedures according to previous reports [46, 55, 56] were followed.

**Table 1:** A repetitive sequence of TrsA, 5S rDNA, 45S rDNA, five bacterial artificial chromosomes (BACs), and four P1 derived artificial chromosomes (PACs) clones were used as probes for FISH.

Chromosomes and arm	Clone ID/Repeats	Markers	Genetic Position^1)^ (cM)	Physical Position ^2)^ (Mb)	Physical location ^3)^ (%)	Chromosomal location ^4)^ (μm)
1S	P0439B06	C50102,S1442,C53447S,C970	5.1	0.5	1.74±0.36	0.68
2S	P0575F10	S2901, E60571	6.9	1.2	4.38±1.15	1.19
3S	OSJNBa0030C11	C1279	11.1	2.1	6.76±1.72	2.28
4L	OSJNBa0070M12	E3142S	129.6	35.0	97.57±0.83	25.82
5S	P0016H04	S12936,S782,S2649	6.6	0.6	3.10±0.86	0.69
6S	TrsA	Tandem repeats			1.44±0.86	0.33
7L	P0496C02	C596	105.7	27.8	90.19±1.58	19.54
8S	OSJNBa12_15D	RG29	3.0		3.34±0.85	0.74
9S	45S rDNA	Ribosomal RNA gene			~18.99± 1.62	3.61
10S	OSJNBb0004A06	R2309,G89B,S21126	4.1	1.9	7.01±0.71	1.21
11S	5S rDNA	Ribosomal RNA gene			39.74±4.91	7.87
12L	TrsA	Tandem repeats			97.52±1.80	20.67
Pericentromere	B1109A06 ^5)^	E2071SA, R1547				

^1)^ Positions are estimated from the end of short arm in cM.

^2)^ Positions are measured from the end of short arm in Mb.

^3)^ Length from the end of short arm shown as %. 100% is considered to be the total length of the chromosome.

^4)^ Length from the end of short arm in μm.

^5)^ BAC clone, B1109A06 contains pericentromeric satellite repeat, CentO and the clone is genetically mapped at 73.4 cM of rice chromosome 1.

### Immunostaining

Anthers were fixed for 30 min in 4% (w/v) *para-*formaldehyde (PFA) in PMEG buffer (50 mM PIPES, 1 mM MgSO_4,_ 5 mM EGTA, 1% glycerol, pH 6.8) [57]. After washing in PBS for 10 min, the anthers were digested for 15 min at 37°C in a mixture of 2% Cellulase Onozuka RS (Yakult Co. Ltd., Tokyo) and 5% Pectolyase Y23 (Seishin Kagaku, Tokyo). The anthers were rinsed in PMEG twice for 5 min each, and squashed on a glass slides in PMEG. After freezing the slides in liquid nitrogen, the coverslips were removed and the slides were air-dried. The slides were incubated for 15 min in a detergent solution (0.5% Triton X-100 in PBS). Slides were washed three times in PBS for 5 min each and blocked with 1% BSA in PBS for 20 min, followed by three PBS washes for 5 min each, and stored until immunostaining. Anti-dimethyl-Histone H3 (Lys9) rabbit immunoaffinity purified IgG (1:200) (Upstate Biotechnology Inc., VA, USA) was used as the primary antibody. Slides were incubated with the primary antibody in a humid dark box at 4°C overnight. After washing the slides in PBS three times for 5 min each, anti-rabbit IgG FITC conjugate (1:80, Sigma, MO, USA) was applied as the secondary antibody. These slides were incubated for 3 hours in a humid dark box at 37°C. After washing the slides in PBS three times for 5 min each, the chromosomes were counter-stained with 1 μg/mL DAPI in Vectashield (Vector Lab.).

## Results

### Identification of individual pachytene chromosomes by FISH

**Fig 1:**
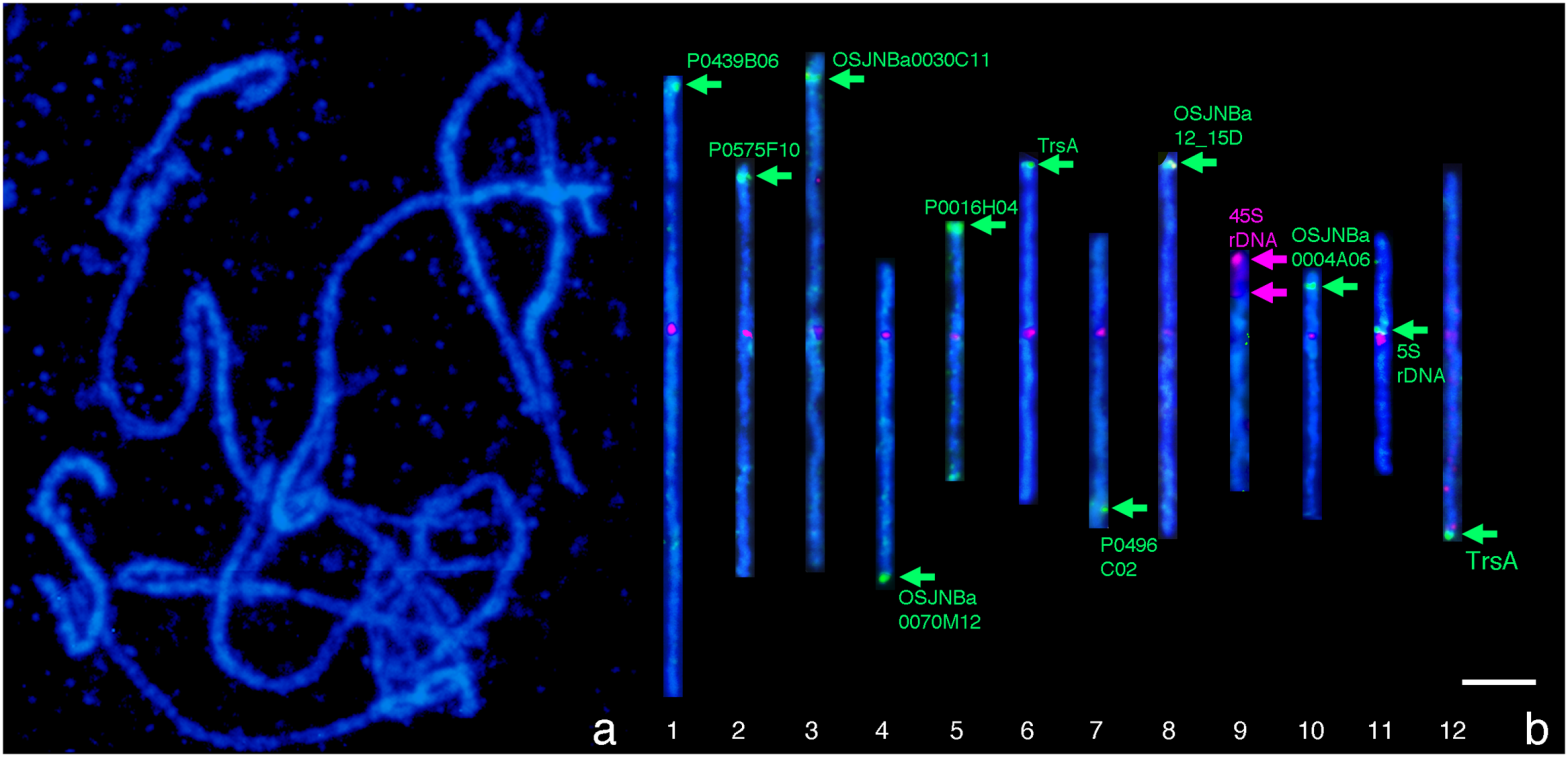
Pachytene chromosome and straightened individual FISH images using the BAC/PAC clones, tandem repeats (TrsA), and rRNA genes on rice pachytene chromosomes. Bar shows 5 μm.

FISH signals from chromosome-specific BAC/PAC clones, a rice tandem repeat TrsA, and 5S and 45S rDNA enabled identification of individual pachytene chromosomes even when they existed alone from the rest of the chromosomes within a complement (Fig 1). Chromosome 1, 2, 3, 5, 8, and 10 were identified based on the presence of the FISH signal on their short arms by the PAC/BAC clones P0439B06, P0575F10, OSJNBa0030C11, P0016H04, OSJNBa12_15D, and OSJNBb0004A06, respectively. Chromosome 4 and 7 were also identified based on the presence of the FISH signals on their long arms by OSJNBa0070M12 and P0496C02, respectively. Chromosome 9 and 11 were identified by the FISH signals of 45S and 5S rDNA, respectively. Chromosome 6 and 12 were both identified by the FISH signals of TrsA. BAC clone, B1109A06, was detected as a red fluorescence (a green fluorescence on only Chromosome 9) at all the centromeric regions. The intensity and size of the centromeric fluorescence signals were significantly varied among the chromosomes and were consistent with the lengths of centromeric nucleotides reported previously [58]. The signals of chromosomes 1, 2, 6 and 11 were relatively stronger and larger, while, for chromosomes 4, 5, 8 and 10 it was weak and small (Fig 1b). The red fluorescence intensity of this study is in relation to CentO content in each centromere [58]. These signals depict the centromeric positions and are definitive landmarks to determine the short and long arm regions of individual rice chromosomes. The signals of BAC clone B1109A06, including the centromeric repeats on chromosomes 9 and 12 seemed to be weaker than the signals on chromosomes 4 and 10 in this particular case (Fig 1b). However, often the intensity of the signals from chromosome 9 and 12 were similar or even stronger than those from chromosome 6 and 7 when observed under a fluorescent microscope. The 5S rDNA was detected as a green signal at the proximal region of the short arm of chromosome 11 as indicated by a green arrow (Fig 1b). Two strong signals for 45S rDNA were detected at the nucleolar organizing regions (NORs) on the satellite, and at the terminus of short arm of chromosome 9, as indicated by the two red arrows. The green signals of TrsA were detected at the distal regions of chromosome 6 and chromosome 12. The fluorescent signal of chromosome 12 is more intense than that of chromosome 6 [59]. The signals from five BAC clones and four PAC clones were observed unambiguously on each rice chromosome. As a result, all the twelve rice pachytene chromosomes were objectively identified.

### Characterization and quantification of the identified pachytene chromosomes by imaging methods

It was previously demonstrated that the condensation pattern (CP) appearing on somatic prometaphase chromosomes can be analyzed by computer imaging [60], but simple application of this method for somatic chromosomes is not suitable for imaging of pachytene chromosomes, because the size and fluorescent intensity of chromomeres vary extremely in the case of pachytene chromosomes. To avoid this problem, CHIAS IV employed a method to evaluate fluorescent intensity relative to adjacent regions, which enables discrimination of the more condensed regions within each chromomere [29]. This study demonstrated the effectiveness of CHIAS IV for detailed structural analysis of pachytene chromosomes. This method was neither technically demanding nor time-consuming, and as a host application, ImageJ is widely used with multiple platforms including Windows, macOS, and Linux.

**Fig 2:**
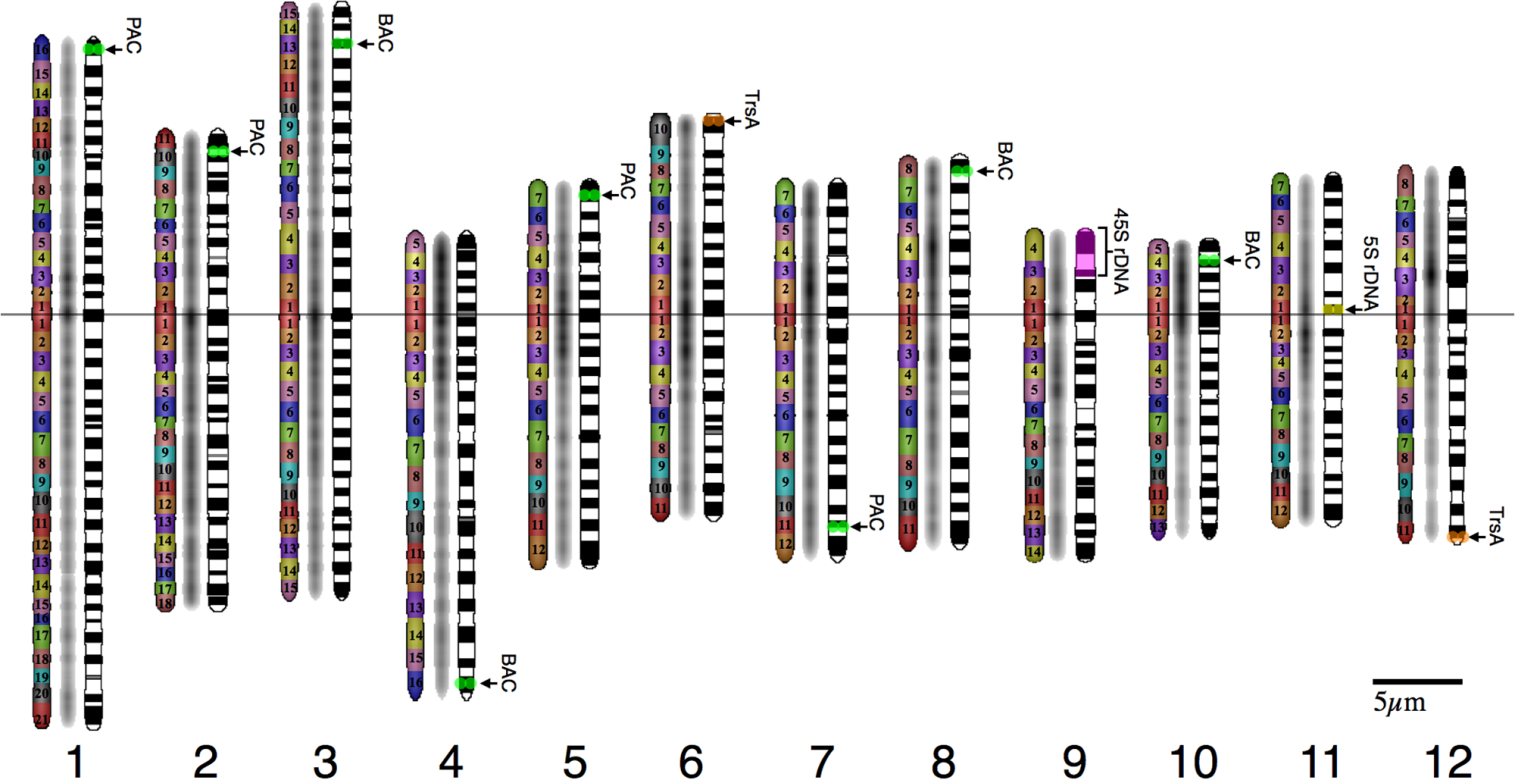
Rice pachytene chromosome map. Distribution of chromomeres with their numbers, fluorescent intensities, and heterochromatic regions with the chromomeres. Left: Chromomere with numbers starting from the centromeric chromomere as 1 to both the terminal regions. Middle: Fluorescence intensity is presented by the graygram. Darker regions correspond to brighter fluorescence intensity. Right: Distribution of condensed regions with the chromomeres. Locations of the BAC/PAC clones (green), TrsA (tandem repeat, orange), and 45S and 5S rDNA (pink and yellow) are indicated on the pachytene chromosome map.

Dimensions of cytological characteristics of the pachytene chromosomes identified by CHIAS IV are presented in Table 2. The quantitative pachytene chromosome map was also developed based on the dimensions, distribution of chromomeres, and their fluorescence intensities (Fig 2). Chromosomes 1, 2, and 3 were associated with a large chromosome group, and were classified as metacentric chromosomes, when Levan’s definition was extended to pachytene chromosomes [61]. Chromosomes 1, 2 and 3 consisted of 36, 28, and 29 chromomeres, respectively, when the centromeric chromomeres at the proximal regions of short and long arms were counted as a single chromomere. In addition, the number of chromomeres indicated the length orders of these long chromosomes as Chromosomes 1, 3 and 2, which was further indicated by nucleotide numbers of the individual chromosomes (Table 2). Chromomeres were distributed along these three large chromosomes commonly lacking intensely stained pericentromeric heterochromatic regions. The signal from OSJNBa0030C11 (11.05 cM, 2.2 Mb) was detected at 6.76±1.72% from the distal end of the short arm of Chromosome 3 (Table 1, Rice Genome Database). The physical length of chromosome 3 was longer than the long arm (Table 2), although the difference was not statistically significant. Chromosome 4 was a subtelocentric chromosome and had 20 chromomeres. Highly condensed heterochromatin was characteristic in the entire short arm and pericentromeric region of the long arm. The heterochromatic region of Chromosome 4 occupied about 17 Mb from the end of the short arm, based on FISH experiment to pachytene chromosome [62] and the revised genome sequence of rice (IRGSP-1.0) [32]. Nucleotide sequence of Chromosome 4 showed that variety of repetitive sequences were located in the heterochromatic regions including the centromeric region except miniature inverted-repeats transposable elements (MITEs), which were located at the euchromatic regions of its long arm [36]. Chromosome 5 was a submetacentric chromosome, being the 6^th^ in the pachytene length order with 18 chromomeres. Highly condensed heterochromatic regions were observed in the pericentromeric regions. Chromosome 6 was a metacentric chromosome consisting of 20 chromomeres, being the 5^th^ in nucleotide number, pachytene and somatic length order. The difference in lengths between short and long arms was small but statistically significant. Highly condensed heterochromatic regions were observed in both the pericentromeric regions. TrsA signal was detected at the terminal position of the short arm of Chromosome 6. Chromosome 7 was a submetacentric chromosome, being the 8^th^ in pachytene length order. It consisted of 18 chromomeres with highly condensed heterochromatic regions in both the pericentromeric regions. Chromosome 8 was a metacentric chromosome, being the 7^th^ in length order as in the case of the somatic chromosomes, consisting of 18 chromomeres. Highly condensed heterochromatic regions were observed at both the pericentromeric regions. Chromosome 9 was a submetacentric chromosome with large NOR regions on the short arm, being the 11^th^ in length order as in the somatic chromosome. A total of 17 chromomeres were visually identified including two chromomeres at the NOR region. The signals for 45S rDNA were reasonably detected at the NOR and the end of the short arm regions (~18.99±1.62%) on Chromosome 9. Although, the NOR region was not firmly condensed through the pachytene stage, the dynamic condensation of the long arm was observed as the pachytene stage proceeded. Chromosome 10 was the smallest submetacentric chromosome showing the shortest in length order as in the somatic chromosome, consisting of 17 chromomeres. Highly condensed heterochromatic regions were observed in the entire short arm and the pericentromeric region of the long arm. Chromosome 11 was a metacentric chromosome, being the 10^th^ in length order, and consisted of 18 chromomeres. Condensed heterochromatic chromomeres were observed in the pericentromeric region. Especially, a large heterochromatic region was observed at the pericentromeric regions in the long arm. Chromosome 12 was a metacentric chromosome, being the 9^th^ in length order, consisting of 18 chromomeres. Condensed heterochromatic region was observed only at the pericentromeric region in the short arm.

**Table 2:** Cytological characteristics of rice pachytene chromosomes with their nucleotide number.

Chrmosome No.**	Chromosome Length (μm)	Short arm** (μm)	Long arm** (μm)	Arm ratio	The number of chromomeres observed***	Nucleotide number (Mbp) *				Compaction of DNA (Mb/μm)			Chromosome length order***,****	
						Total	Short arm	Long arm	Arm ratio	Total	Short arm	Long arm	pachytene chrs.	Somatic chrs.
1	39.00±5.47^a^	15.67±3.08^a^	23.33±2.54^a^	1.49±0.13	36	43.27	16.93	26.34	1.56	1.11	1.08	1.13	1	1
2	27.13±1.51^c^	10.47±1.07^bc^	16.67±0.61^b^	1.59±0.14	28	35.94	13.71	22.23	1.62	1.32	1.31	1.33	3	3
3	33.67±1.36^b^	17.53±1.58^a^	16.13±1.45^bc^	0.92±0.15*****	29	36.41	19.59	16.83	0.86	1.08	1.12	1.04	2	2
4	26.47±2.15^cd^	4.87±0.25^e^	21.60±2.23^a^	4.44±0.57	20	35.50	9.86	25.64	2.60	1.34	2.03	1.19	4	4
5	22.13±2.19^cdef^	7.80±0.61^d^	14.33±1.70^bcd^	1.84±0.15	18	29.96	12.51	17.45	1.40	1.35	1.60	1.22	6	8
6	22.93±1.26^cde^	11.20±0.75^b^	11.73±0.53^d^	1.05±0.03	20	31.25	15.44	15.80	1.02	1.36	1.38	1.35	5	5
7	21.67±1.94^def^	7.73±1.02^d^	13.93±1.20^bcd^	1.80±0.21	18	29.70	12.08	17.62	1.46	1.37	1.56	1.26	8	10
8	22.13±2.13^cdef^	9.00±1.21^bcd^	13.13±1.64^bcd^	1.46±0.27	18	28.44	12.95	15.49	1.20	1.29	1.44	1.18	7	7
9	19.00±1.45^ef^	5.00±0.32^e^	14.00±1.19^bcd^	2.80±0.16	17	23.01	2.90	20.12	6.94	1.21	0.58	1.44	11	11
10	17.27±1.86^f^	4.53±0.42^e^	12.73±1.53^cd^	2.81±0.25	17	23.21	8.20	15.01	1.83	1.34	1.81	1.18	12	12
11	20.07±3.46^ef^	8.27±1.60^cd^	11.80±2.30^d^	1.43±0.27	18	29.02	12.26	16.76	1.37	1.45	1.48	1.42	10	9
12	21.20±0.62^def^	8.33±0.96^cd^	12.87±1.28^cd^	1.54±0.36	18	27.53	11.93	15.60	1.31	1.30	1.43	1.21	9	6

*) Nucleotide number of each total chromosome is based on the IRGSP-1.0 database (Kawahara et al., 2013). The lengths of short and long arms are determined by the midpoint of the annotated centromere in the IRGSP-1.0.

**) Chromosome number and arm assignment is based on the RAP (Rice Annotation Project).

***) The chromomeres were analyzed using five samples from each chromosome.

****) Fukui & Iijima (1991).

*****) Arm ratio based on the data of the short and long arm is 1.09±0.18.

### Comparative analyses of the four maps

**Fig 3:**
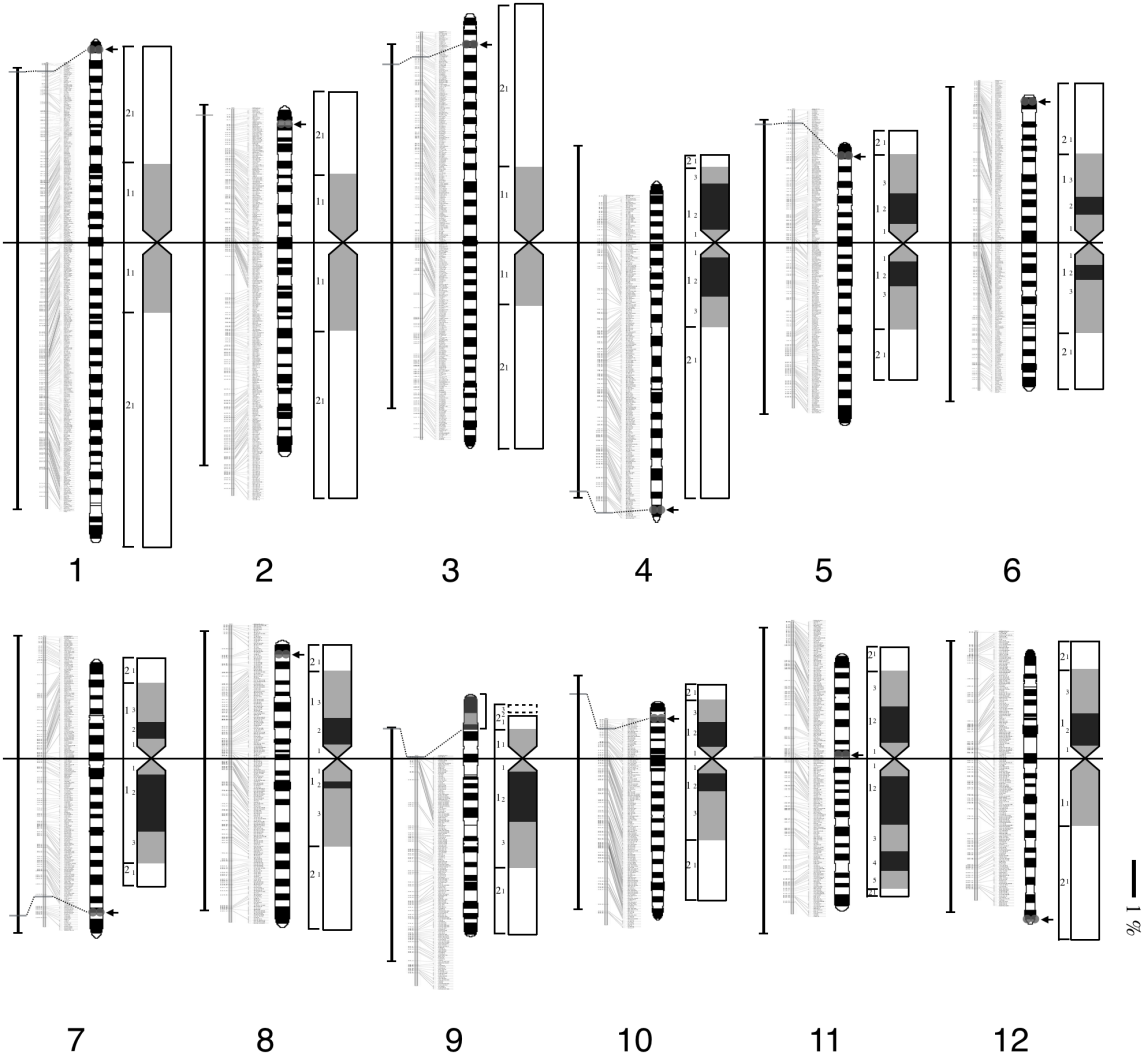
Integration of three rice maps; from left to right: Nucleotide numbers, linkage map, meiotic pachytene chromosome map, and mitotic prometaphase chromosome map. The length ratios among 12 chromosomes were adjusted to the ratios of pachytene chromosomes. Bar shows 1% region for all the maps. Designation of the chromosome number and short/long arms of somatic chromosomes follows the IRGSP-1.0 database32. As a result, chromosome number and assignment of long and short arms sometimes do not follow their actual length order.

Four different rice chromosome maps; physical map (IRGSP-1.0) [32], the linkage map (Data source: RGP Public Data, http://rgp.dna.affrc.go.jp/E/publicdata/geneticmap2000, Map figure: Oryzabase, https://shigen.nig.ac.jp/rice/oryzabase/marker/about), somatic prometaphase chromosome map [1, 8] and the pachytene chromosome map developed, were visually compared (Fig 3). In Fig 3, the length of each chromosome was drawn as the relative length (%) to its total chromosome length. Comparison of nucleotide length, and the three different maps provide interesting and deep insights into rice chromosome structure.

First, Fig 3 depicts that the three maps with different bases present very similar tendency among them. This point is clearly illustrated by the fact that the designations of short and long sides are the same for all the four representations of rice chromosomes except for Chromosome 6. This means that the nucleotide number primarily determines all the genetic recombination values and chromosome lengths at mitosis and meiosis. Second, rice chromosomes were divided into two types—large and small chromosomes as mentioned earlier [1]. The larger type consists of chromosome 1, 2 and 3, without a heavily condensed region at the prometaphase stage [1]. The analysis of pachytene chromosomes, however, revealed that the chromosome 2, the third longest chromosome is relatively condensed at the pachytene stage. Thus, although rice chromosomes are grouped into large and decondensed, and small and condensed, to which the chromosome 2 belongs, based on the type of division. Third, chromosome 11 has two heavily condensed regions on the long arm, however, there is no specific structure in the pachytene chromosome. Thus, the structures of somatic and pachytene chromosomes could be different, although the higher order structures of somatic and pachytene chromosomes are not known yet.

The lengths of linkage maps, pachytene chromosomes, somatic chromosomes, numbers of repetitive sequences, and genes of each chromosome arm, were subjected to regression analysis of DNA length of rice genome (S1A–S1F Fig). In the case of length of linkage maps (A), the number of repetitive sequences (D), and the genes (E), null hypothesis that the intercept of the regression line on zero is rejected statistically. Accordingly, the intercept determines the regression line on non-zero (a solid line in A, D, E). The 95% confidence limits of the regression line are drawn as a dotted line, and the 95% prediction interval, including the residue data are drawn as dotted lines. All of the chromosome arms are positioned on the inside of the 95% prediction interval. This result indicates that the genome DNA length is directly proportional to the length of linkage map, the number of repetitive sequences and the genes (A, D, E).

Examination of each of the FISH signals in the figure between the nucleotide and linkage map length (A), revealed that five of seven FISH signals were located inside the 95% prediction intervals (rhomboidal mark in A). In the case of the association of somatic chromosome length (B) and pachytene chromosome length (C) to the nucleotide numbers, the null hypothesis of the regression line on zero is not rejected. In addition, null hypothesis of the regression line on zero is not rejected in the regression analysis of somatic and pachytene chromosome lengths (F). Thus, the regression line is calculated as a straight line passed through origin. Then the 95% confidence intervals (dashed line) and the 95% prediction intervals (dotted lines) are drawn using the same procedure.

All the chromosome arms are positioned within the 95% prediction interval area. The specific chromosome condensations are different among chromosome arms, as the range is 0.09–0.18 μm/Mb in somatic chromosomes, and 0.49–1.73 μm/Mb in pachytene chromosomes. It is possible that chromosome arm length could be determined to be directly proportion to the nucleotide number, for instance, by overviewing the entire somatic and pachytene chromosome arms as shown in S1B and S1C Fig, respectively. There are no characteristic differences between somatic chromosomes and pachytene chromosomes in regard to their lengths (F), although chromosome 10S and 4S arms are highly condensed in comparison to the other chromosomes.

**Fig 4:**
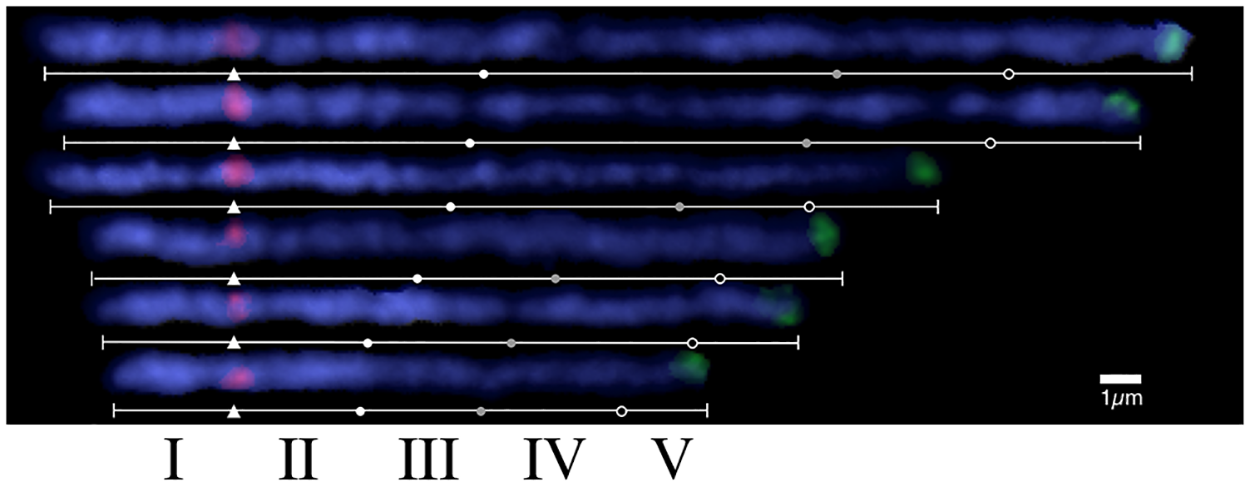
Differential condensation of chromosome 4 during the pachytene stage. Chromosome 4 is represented by four different marks of centromere (Δ, red fluorescence), distinctive regions of long arm (white, gray, and black circles), and telomere (green fluorescence) in order to reveal the differential condensation dynamics occurring at all chromosomal regions.

Fig 4 shows six straightened pachytene Chromosome 4 arranged in the length order during pachytene stage. Red and green fluorescence signals show centromere and terminal position of the long arm of Chromosome 4, respectively. White triangles indicate centromeric positions (Δ) and the three tertiary constrictions are indicated by white, gray, and blank circles from the proximal region of the centromere. By using these five landmarks, pachytene Chromosome 4 is divided into five regions, one is whole short arm (I), and four regions (II-V) belong to long arm. This compartmentalization enables revealing the local dynamics in condensation ratio. The maximum condensation was observed at the regions III with 65.6% reduction from the longest stage, and the least condensation was the region IV with 15.8% reduction, respectively. It is thus, obvious that even among the dispersed regions (III, IV and V), condensation rates during the pachytene stage are not uniform among these.

### Histone H3K9 dimethylation on rice pachytene chromosomes

**Fig 5:**
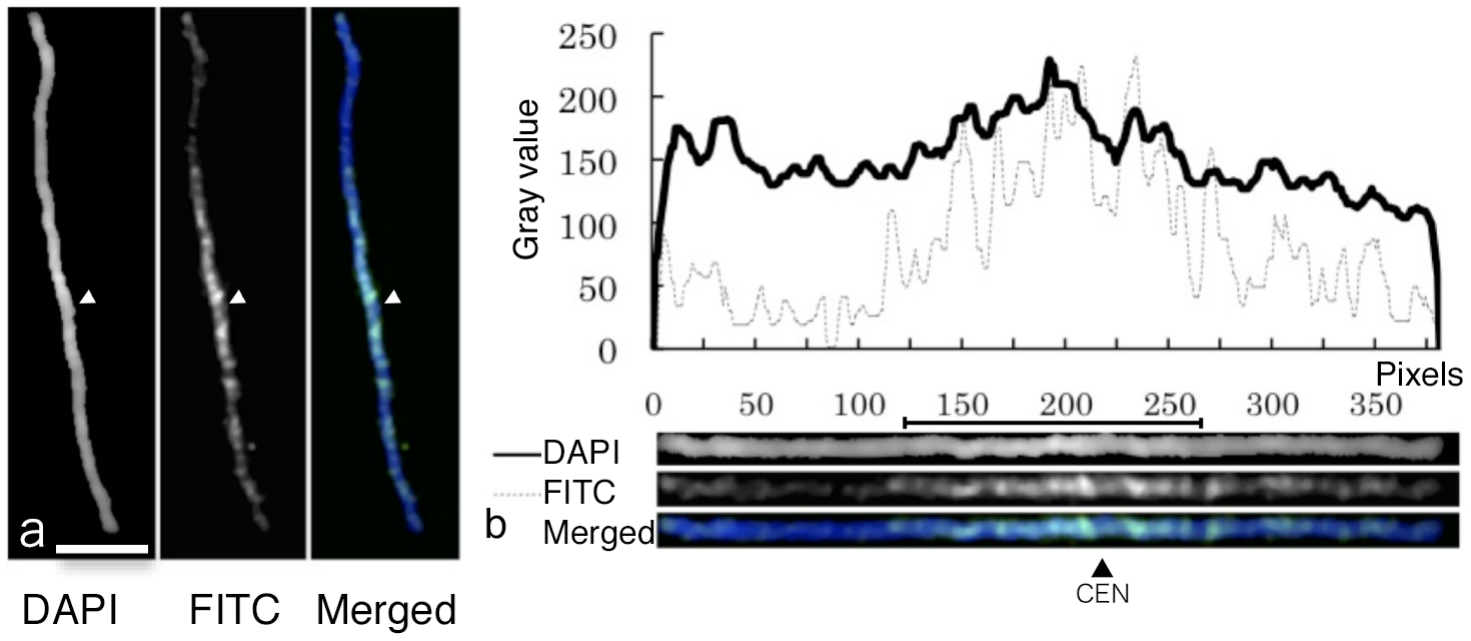
Fluorescence patterns of pachytene chromosome with DAPI and immunostaining of histone H3K9 dimethylation. a: Chromosomal Image. Bar shows 5 μm. b: Two curves are the fluorescence profiles indicating fluorescence intensities of DAPI-stained chromosome and immunostaining of H3K9me2. Straightened chromosome images show DAPI, FITC (H3K9me2), and merged images. Arrowheads indicate centromeres.

Finally, we examined the histone H3K9me2 distribution pattern on the pachytene Chromosome 11 by immunostaining using the anti-histone H3K9me2 antibody. Histone H3K9 dimethylation (H3K9me2) is one of the well-known heterochromatin markers in plant chromosomes [43]. H3K9me2 is known as the marker indicating inactivation of the gene [63]. The fluorescent signals were specifically located at heterochromatic regions of individual chromomeres (Fig 5a). There are seven and twelve chromomeres on respective short and long arms. Among 19 chromomeres in total, 16 show prominent FITC signals at the condensed regions within the chromomeres. The peaks of fluorescence profiles of H3K9me2 and DAPI-stained chromomeres of these 16 chromomeres also corresponded to each other (Fig 5b), indicating the tendency of gene distributions along whole chromosome. We did not use the centromere repeat FISH method because morphological identification of centromeric positions was easy to do in this case. Centromere repeat FISH would be an effective method to identify the position of rice chromosomes [20].

## Discussion

### Integration of physical, linkage and chromosome maps

In this study, we developed a quantitative pachytene chromosome map and explored the integration of the physical, linkage, and two chromosome maps. The integration revealed the following characteristics of rice chromosomes (Fig 3). 1) The chromosome lengths are basically determined by the DNA length. 2) Rice chromosomes consists of two types of chromosomes, large and decondensed chromosomes, and small and condensed chromosomes. Chromosome 1 and 3, are large decondensed chromosome, while Chromosome 2 is the condensed chromosome 3) Chromosome 11 is the somatic chromosome that has two heavily condensed regions, but no condensed regions in the pachytene chromosome. Short arms of Chromosome 9 have lower recombination values caused by the abundant ribosomal RNA genes.

We analyzed detailed cytological characteristics of rice pachytene chromosomes, and formulated them into the pachytene chromosome map with the positions of chromosome-specific BAC/PAC clones, 45S and 5S rDNA, and a TrsA. We used late pachytene chromosomes for image analysis, because with late pachytene chromosomes it was relatively easy to obtain individual chromosome images, and the regions of individual chromomeres were relatively distinct compared to the other pachytene stages. The distinct chromomeres unique to each chromosome were arrayed in a linear manner along the rice chromosomes and some of the chromomeres located at highly condensed heterochromatic regions (Fig 2). These chromomere distribution patterns are quite different from *Arabidopsis*, where heterochromatic regions are exclusively confined to pericentromeric regions (chromocenter) of all the pachytene chromosomes with the rest of euchromatic regions in both the terminal regions [64]. Chromomere distribution through rice chromosomes served not only as good landmarks to distinguish each chromosome, but also as the local markers within a chromomere useful to address each fine region. It should be pointed out that the chromomeres were distributed more or less evenly on pachytene chromosomes, however, recombination values were quite different between the regions. Non-random distribution of recombination values across chromosomes has been observed in many plant species including rice, and recombination is usually suppressed in heterochromatic regions [12, 37, 65, 66], although this tendency was not very clear in our case.

Genes on pericentromeric regions would be difficult to precisely map on a linkage map, and might be difficult for breeders to use them in breeding programs because of little information on recombination values. Therefore, FISH method is valuable to determine the precise locations of target genes on pachytene chromosomes. The mapping resolution of FISH depends on species, cytological targets (e.g., interphase nuclei, somatic and meiotic chromosomes, and DNA fibers) and structure of chromosomes [67]. Previous reports showed that the resolution of rice pachytene FISH is ~100 kb in euchromatic region [68], while the resolution of somatic chromosomes is 2–5 Mb [69, 70]. In tomato, the resolution of pachytene FISH is 120 kb and 1.2 Mb in the euchromatic and heterochromatic regions, respectively [42]. In *Arabidopsis*, pachytene FISH can resolve 60 and 140 kb in the euchromatin and heterochromatin regions, respectively. It is also reported that early pachytene chromosomes have higher resolution, which are two times longer than the late ones [38]. The pachytene map developed in this study serves as the basic map of FISH signals with the resolution of 43–136 kb on average, but has a similar resolution to *Arabidopsis*. For this research, the condensation of Chromosome 4 during pachytene stage changed dynamically based on the pachytene chromosome condensing stages. This means FISH markers on chromosome are important for the ideogram to develop the accurate genetic information.

The linkage and physical maps are beneficial to determine the positions of genes and specific DNA sequences. Linkage map based on recombination values has limitation, because it is assumed that genetic recombination occurs at random across all the chromosomes and chromosomal regions. Therefore, the integration of linkage and chromosome physical maps is important for the accurate positioning of genes and specific DNA sequences [12]. Overall, chromosomal condensation showed similar patterns between them. For example, Chromosome 4 at both of prometaphase and pachytene stage had highly condensed heterochromatic regions at the entire short arm, and the proximal region of long arm. Pachytene Chromosomes 9 and 11, having condensed chromomeres on their long arms also had heterochromatic regions on the long arm of the prometaphase chromosome, which could be related to the positions of rDNA. Recombination ratios relatively co-lineate along the pachytene chromosome length. Chromosome 1 and 3 whose DNA densities were relatively low in pachytene stage have no heavily condensed regions in prometaphase chromosomes [1, 8].

### Formation of heterochromatin

In general, heterochromatin is composed of repetitive sequences such as transposable elements and other types of repetitive elements [71]. Heterochromatin plays a significant role in repressing activities of genes and transposable elements. Large blocks of heterochromatin surround functional chromosome regions as centromeres, whereas, smaller heterochromatic domains are interspersed throughout the chromosome in rice. DNA methylation, histone modification, and recruitment of specific protein complexes are known to be responsible for heterochromatin formation and maintenance [72]. In *Arabidopsis*, histone H3K9me2 has been known as one of the epigenetic markers of heterochromatic regions in somatic nuclei and pachytene chromosomes [73]. This heterochromatin specific histone modification is partially conserved among plant species. Houben et al. [74] proposed two types of distribution patterns of histone H3K9me2 in somatic nuclei, depending on their genome size. The species with a genome size of 500 Mbp or less showed high level of histone H3K9me2 at heterochromatic regions, whereas the species with a larger genome size, such as, maize showed a dispersed distribution pattern of histone H3K9me2 throughout the nucleus. In maize pachytene chromosomes, histone H3K9me2 marked both the euchromatin and heterochromatin [8] that is different from maize mitotic nuclei [74], and from *Arabidopsis* pachytene chromosomes [73]. The rice genome (390 Mb genome size) has no distinct heterochromatic chromocenters in the nucleus like *Arabidopsis*, but has heterochromatic regions in pachytene chromosomes. The signals of rice histone H3K9me2 were dispersed all over the nucleus with strong foci, but were enriched at heterochromatic regions in pachytene chromosomes, indicating the correlation between H3K9me2 and heterochromatin in rice. In this study, we also compared fluorescent profiles of DAPI-stained chromomeres and H3K9me2 signals, and found both the peaks were located at the same positions. This result suggests that H3K9me2 likely plays important roles in formation of chromomeres in rice pachytene chromosomes. A mutant of *Caenorhabditis elegans* with reduced H3K9me2 is defective for meiotic recombination and chromosome segregation [75] and a study reported the impact of histone H3 acetylation on meiotic crossing over formation in *Arabidopsis* [76]. H3K9me is believed to be an obligate characteristic of heterochromatin, and the presence of H3K9me at specific loci in *Arabidopsis* correlates with heterochromatic silencing [77].

### Chromosome structure and transcriptional activity

Now an interesting question arises as to how chromosomal structure is associated with transcriptional activity. Several studies showed that transcriptionally active regions coincided with euchromatic regions in pachytene chromosomes [78]. More interestingly, uneven patterns of transcriptional activity in the heterochromatic regions of the entire short arm and the proximal part of long arm of Chromosome 10 was consistent with chromomeric patterns: the transcriptional activity is reduced in condensed regions in each chromomere, but not in their flanking decondensed regions [78]. Transcriptional activity associated with chromosomal architecture seems to be stage- and tissue-specific, and depends on the developmental stages and tissues [62, 79]. It is provable that the gene expression is related to chromosomal architecture, especially the degree of its condensation, which is regulated by histone modification. For example, it is reported that vernalization [80, 81], submergence-inducible genes expression [82] and osmotic/salt stress [83] are controlled through histone modifications.

## Conclusions

Gene-based chromosome research combined with cytology is still in the developing stage. A comprehensive view of interactive relationships among epigenetics, chromosomal structure, transcriptional activity relating to cell differentiation and development, has not been fully uncovered yet. It is interesting to know what is a general- or specific-role of heterochromatin or euchromatin from a genetic point of view, as well as how chromosome structure is actually organized and involved in the transcriptional activity of genes. It is believed that the high-resolution chromosome map, including the information of histone methylation region, and the chromatin condensation can contribute to plant genetics. Detailed quantitative analyses of chromosomes at both, the somatic prometaphase and pachytene stages are necessary to shed light on knowledge gaps in the relationship between chromosomal structure and transcriptional activity, as well as plant epigenetics. CHIAS was used for the chromosome study of various cultivated crops such as rice, sugarcane, soybean, red clover and *Brassica* [56, 84, 85]. We believe that this study would contribute to a better understanding of rice chromosomes, and possibly lead to further discovery of chromosome functions and structure.

## Supporting information

S1 Fig Comparison of various lengths with nucleotide physical length. Black dots indicate individual chromosome arms. 1) Physical length, linkage value, and somatic length are based on IRGSP 1.0 (Kawahara et al., 2013) [32], RGP (http://rgp.dna.affrc.go.jp/E/publicdata/geneticmap2000/index.html), and Fukui and Iijima (1996) [1], respectively. 2) Values of repeat units and gene length are obtained from IRGSP 1.0 database (Kawahara et al., 2013) [32]. **) Lengths in 9S arm are not included satellite region (ribosomal RNA gene).
(TIF)
