# Supplementary figures and images for: Development of a quantitative pachytene chromosome map and its unification with somatic chromosome and linkage maps of rice (Oryza sativa L.)
Source: PLoS One. 2018 Apr 19;13(4):e0195710. doi: 10.1371/journal.pone.0195710 (PMC5908146; doi:10.1371/journal.pone.0195710)

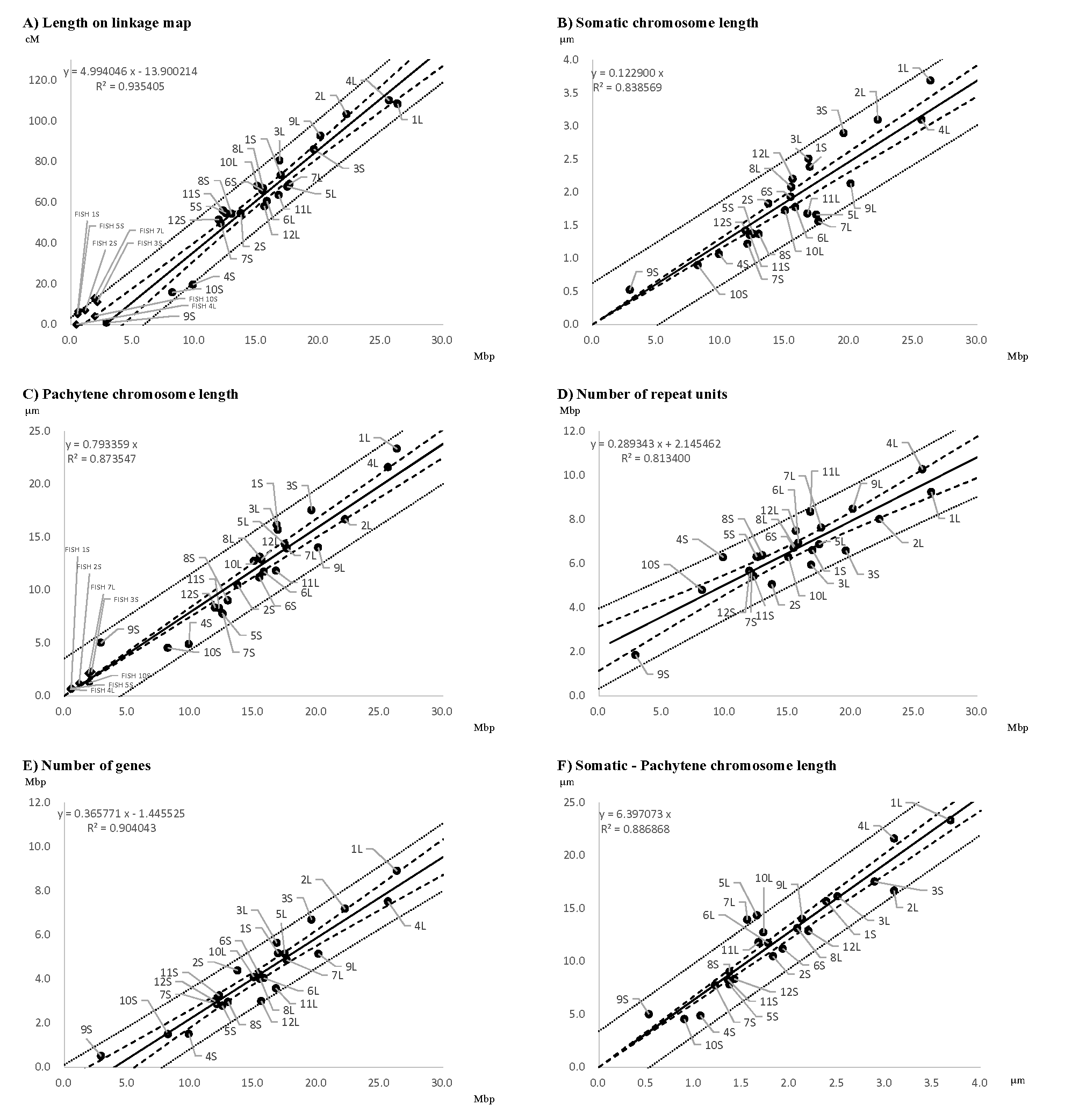

Supplement: S1 Fig — Black dots indicate individual chromosome arms. 1) Physical length, linkage value, and somatic length are based on IRGSP 1.0 (Kawahara et al., 2013) [32], RGP (http://rgp.dna.affrc.go.jp/E/publicdata/geneticmap2000/index.html), and Fukui and Iijima (1996) [1], respectively. 2) Values of repeat units and gene length are obtained from IRGSP 1.0 database (Kawahara et al., 2013) [32]. **) Lengths in 9S arm are not included satellite region (ribosomal RNA gene). (TIF) [file pone.0195710.s001.tif]
